# Improved Object Localization Using Accurate Distance Estimation in Wireless Multimedia Sensor Networks

**DOI:** 10.1371/journal.pone.0141558

**Published:** 2015-11-03

**Authors:** Yasar Abbas Ur Rehman, Muhammad Tariq, Omar Usman Khan

**Affiliations:** 1 NUSys Lab/ National University of Computer and Emerging Sciences (NUCES), Peshawar, KPK, Pakistan; 2 Department of Electrical Engineering/City University of Science and Information Technology, Peshawar, KPK, Pakistan; Nankai University, CHINA

## Abstract

Object localization plays a key role in many popular applications of Wireless Multimedia Sensor Networks (WMSN) and as a result, it has acquired a significant status for the research community. A significant body of research performs this task without considering node orientation, object geometry and environmental variations. As a result, the localized object does not reflect the real world scenarios. In this paper, a novel object localization scheme for WMSN has been proposed that utilizes range free localization, computer vision, and principle component analysis based algorithms. The proposed approach provides the best possible approximation of distance between a wmsn sink and an object, and the orientation of the object using image based information. Simulation results report 99% efficiency and an error ratio of 0.01 (around 1 ft) when compared to other popular techniques.

## Introduction

The accessibility of low cost and low complexity multimedia hardware like cameras and microphones has allowed for the transformation of existing Wireless Sensor Networks (WSN) to Wireless Multimedia Sensor Networks (WMSN). Although the variability in services provided by recent WMSN have surpassed traditional WSN, there are certain constraints which are imposed by the properties inherited from traditional WSN [1–3]. These constraints include limited processing capabilities, battery power, bandwidth, and on-board memory. Since, both WSN and WMSN involve battery powered devices, the need for energy conservation to prolong sensor lifetime is an important design parameter when modeling and designing protocols, algorithms and services for these networks. One important technique that is used in designing these services is the localization of individual sensor nodes or objects within the network [4]. Amongst the various node localization techniques, range free methods have gained wide-spread attention due to their ability to localize devices with an acceptible error tolerance without the need for any specialized hardware component [5, 6]. Object localization involves estimation of an object’s position within a network as it traverses a path in-between individual WSN nodes [7].

A key problem in WMSN is the isolation of objects from image backgrounds [8]. Most approaches assume static nodes and thus rely on simple background subtraction based methods [9–12]. Another approach takes into account fusion of information from multiple WMSN nodes to compensate for localization error [13]. In real-time, however, the assumption of static nodes can lead to false results due to uncontrollable environmental variations. Another source for false detection can be attributed to lack of consideration to the geometry and orientation of an object. This false information is then eventually conveyed to sink nodes. The orientation of a node with respect to an object is mostly unpredictable and involves the fusion of disparate WMSN nodes [14].

The central theme of this paper is to effectively isolate and locate objects from images by fusing range free techniques with machine learning and computer vision algorithms. The objective would be to increase object localization accuracy in the real-world from images received at the sink node by utilizing coordinate information of sensor nodes using Principle Component Analysis (PCA) and computer vision algorithms. The energy consumption footprint of this method will also be minimized in order to prolong the WMSN lifetime. The proposed method will be analyzed and compared to other existing techniques.

The rest of this paper is organized as follows: The related work discusses the state of the art work done in range free localization techniques and object localization techniques. The methadology section defines the proposed methodology of object localization in a localized WMSN. The results are discussed and analyzed in simulation results section. The paper is then concluded with a direction to the future work.

## Related Work

Since WMSN essentially involve WSN with multimedia equipped devices [15], it can therefore be assumed that they can inherit traditional range free based localization algorithms. This assumption inspires us to review some of related state of the art work in the area.

The basic aim of range free localization algorithms is to locate individual sensor nodes by utilizing existing resources without depending on additional hardware infrastructure [16]. This localization process is completed in three steps; determining the relative distances between individual nodes, approximating position of nodes by solving a set of linear equations simultaneously, and finally, refining the position by utilizing position information from neighboring nodes [17]. For efficiency, the localizing scheme must be robust and energy efficient in areas of low sensor density, or when obstacles are present between sensing nodes. Likewise, the scheme must also hold against un-determinant nodes [18–20]. However, since WSN deployments are usually random, therefore anisotropic patterns and holes can pose a challenge. For these deployment related constraints, detour path angular information (DPAI) based localization can be used [21].

For object localization, computer vision based algorithms are predominantly used for the purpose of detection, recognition, and tracking [22–25]. As can be anticipated, vision based algorithms in a distributed setup would not only involve processing overhead, but also constrain image transmission on limited bandwidths. As such, data compression would be natural [26]. The processing overhead would be associated with algorithmic complexity. For tracking based applications, continuous capture of a target would entail a significant energy footprint. Various solutions exist to minimize this footprint; ranging from rotational camera sensors [27] to time-stamped varying information capture using single static cameras [28]. Another approach to reduce transmission latency and conserve energy is the adoption of cluster based approach for communication with the aid of Kalman filters [29]. As such, each cluster will track objects using cooperative communication between cluster elements in order to aggregate data.

The real-world coordinates of objects can be estimated using image based coordinates using intrinsic and extrinsic properties of cameras [28]. However, numerous difficulties arise in this transformation process, for instance, the disturbance of camera positions due to strong winds, the presence of moving artefacts, or shadowing effects. It is, therefore, not sufficient to send image based information to sink node’s but object meta-data from the image must also be conveyed [30]. To extract this information, a straight-forward approach based on frame differencing can be possible [9–12] assuming static nodes. However, in the scenarios just mentioned, this method will be less efficient. To cope with this problem, this paper proposes to localize objects in WMSN using image information received from different nodes at the sink node. The orientation of the object will then be carried out with respective to the sink. The proposed methodology utilizes a fusion of range free localization, PCA, and computer vision based algorithms to accurately localize a target object while respecting energy constraints of the WMSN nodes.

## Methodology

Consider a heterogeneous WMSN network with *m* multimedia and *s* sensor nodes deployed randomly in a field. To localize an object *o* traversing a path inbetween the nodes, it is important that the other WMSN nodes, including the sink node are also aware of their positions. The WMSN node positions are localized using the DPAI procedure [21] by obtaining location information of anchor nodes in the network. Once the nodes are localized, the sink node floods its unique identity to all nodes in the network. Upon receipt of an identity packet, WMSN multimedia nodes will start the object localization process. This process is illustrated in Algorithm 1.

**Algorithm 1**: In-Node Process

**Input**: *V* = {*v*_1_…*v*_*n*_}: Set of WMSN nodes with unknown location

*D*_*x*_*d*_,*y*_*d*__: Sink node location

*F*_*i*_: Frame captured by *v*_*i*_ node

**Output**: *V*_*X*,*Y*_ = {*v*_1_*x*,*y*__, *v*_2_*x*,*y*__, …, *v*_*n*_*x*,*y*__}: Localized nodes with location information

*LL*1_*i*_: Low-Low level 1 sub-band corresponding to frame *i*

**Node Localization** localize()

**for** *i* = 1 → *n* **do**

1   *calculate*(*x*, *y*) ∈ *v*_*i*_; Using **DPAI** [21]

**Frame Capture & Transmit** captureTransmit()

**Fig 1:**
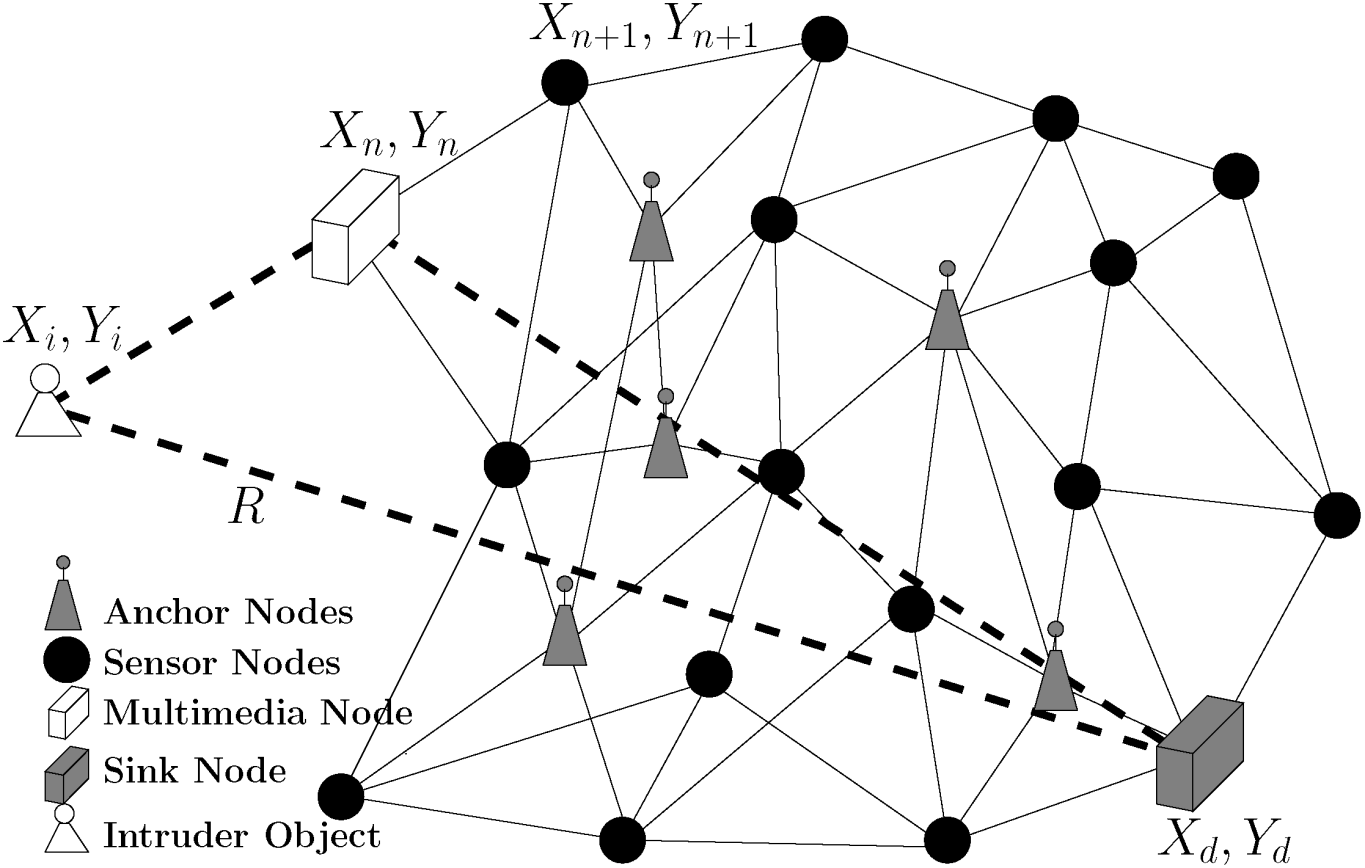
WMSN Network with *m* multimedia nodes and *s* sensor nodes.

1   *captureF*_*i*_;

2   *extract*(*LLI*_*i*_, *Sub* − *Band*);

3   *packetize*{*LLI*, *v*_1_*x*,*y*__};

4   *transmit*{*LLI*, *v*_*i*_*x*,*y*__};

An example scenario is depicted in Fig 1, where a multimedia node with location (*X*_*n*_, *Y*_*n*_) captures an image of the scene containing a target object. This image is then decomposed into four multi-resolution images using 2D Discrete Wavelet Transform (2D-DWT). Of these decomposed images, only the coarse level coefficient image, i.e., the Low-Low level 1 (LL1) sub-band is transmitted to the sink using a multi-hop route. The selection of the LL1 sub-band has multiple advantages as it entails minimum processing, storage, and transmission energy. The small size is thus ideal when considering the limited bandwidth properties of the WMSN. The sink-node, upon receipt of the LL1 sub-band image, performs post-processing using computer vision algorithms aided by PCA technique. This process extracts an object from the received image, and is shown in Algorithm 2.

**Algorithm 2**: Object Localization at Sink Node

**Input**: *V*_*X*,*Y*_ = {*v*_1_*x*,*y*__…*v*_*n*_*x*,*y*__}: Localized nodes along with location information

*LL*1_*i*_: Low-Low level 1 sub-band corresponding to node *i*

**Output**: *d*_*min*(*i*)_: Minimum distance to object from *v*_*i*_ node

*C*_*D*,*O*_: Distance from *D*_*Xd*,*Yd*_ to object *O*_*i*_

*β* ^*T*^: Angle between object and sink node

**Select Object Close to Node** *v*_*i*_ selectObject()

**for** *i* = 1 → *n* **do**

1    extract *O*_*i*_(*LL*1_*i*_);

**if** *O*_*i*_ = = 1 **then**

2    *v*_*i*(*x*,*y*)_ = *v*_*i*(*Xs*,*Ys*)_;

3   Calculate *B*_*D*,*N*_;

4   Calculate *O*_max(*i*)_;

**Compute** *D*_min_ **from** *v*_*i*_ **to** *O*_*i*_ computeDMin()

1    Calculate *P* = *A* − *μ*;

2    Calculate *U* = *P* ^*T*^ ⋅ *EGV*_1, …, 20_;

3    Calculate *D*_*i*_ = *normF*_*i*_ − *U*;

4    Calculate *D*_min*i*_ = {*D*_*i*_};

5    *A*_*O*,*N*_ = *D*_min*i*_;

**Compute Orientation & Distance from Object to Destination**

DestinationOrientation()

1   Calculate $C_{D,O}\sqrt{A_{O,N}^{2}+B_{D,N}^{2}}$;

**if** *C*_*D*,*O*_ > *B*_*D*,*N*_ **then**

2     $\beta^{T}={\text{tan}}^{-1}\frac{A_{O,N}}{B_{D,N}}+{\text{tan}}^{-1}\frac{dp^{\prime }}{db^{\prime }}$;

**else**

**if** *C*_*D*,*O*_ < *B*_*D*,*N*_ **then**

3      $\beta^{T}={\text{tan}}^{-1}\frac{A_{O,N}}{C_{D,O}}+{\text{tan}}^{-1}\frac{dp^{\prime }}{db^{\prime }}$;

**else**

**if** *C*_*D*,*O*_ = = *B*_*D*,*N*_ **then**

**Fig 2:**
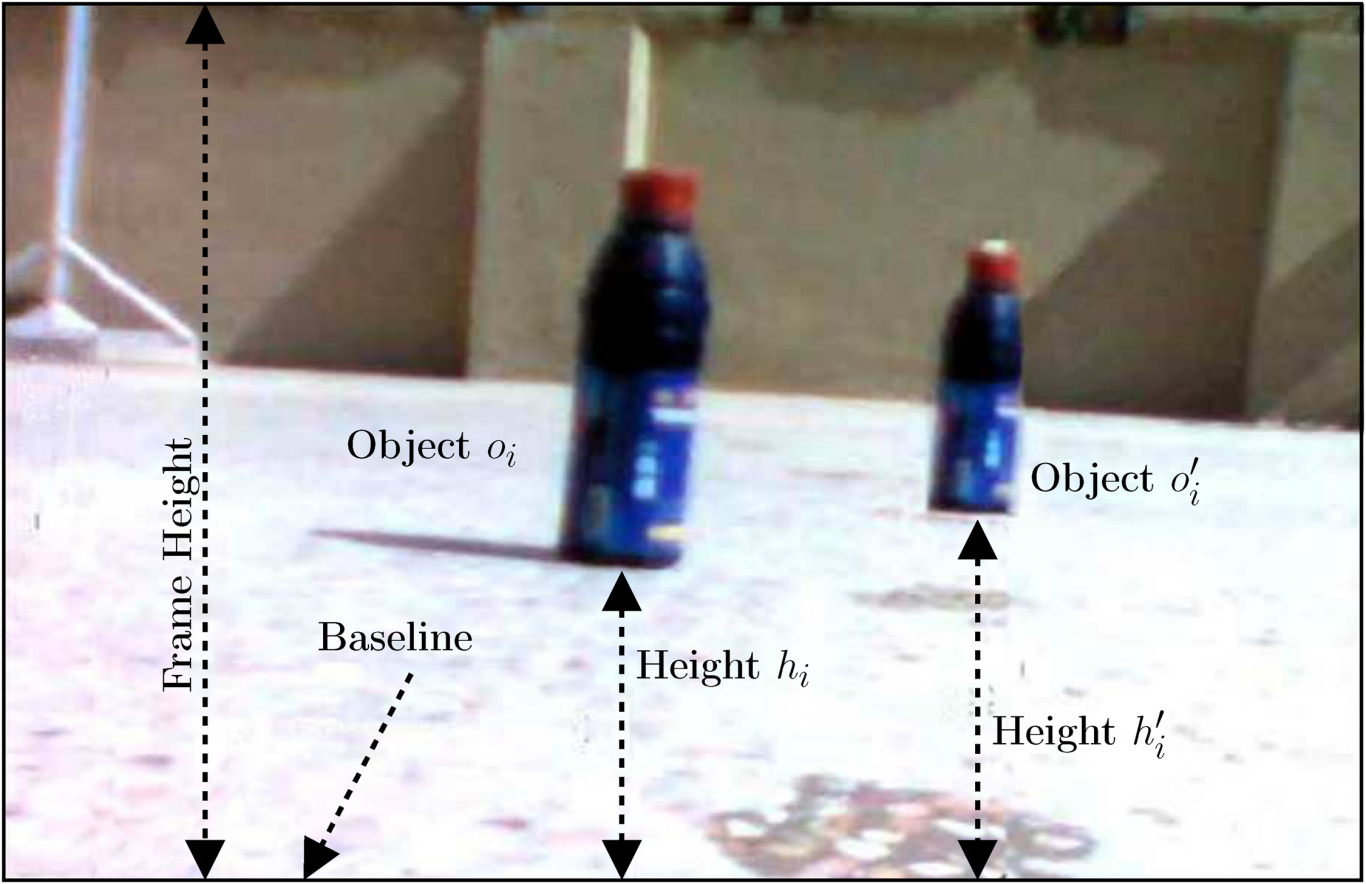
Object *o*_*i*_ has maximum size and minimum height *h*_*i*_ as compared to object *o*′_*i*_ with height *h*′_*i*_.

4       $\beta^{T}={\text{tan}}^{-1}\frac{dp^{\prime }}{db^{\prime }}$;

To obtain location information of the object, it is necessary to estimate it’s distance from the WMSN node. This distance is obtained by preparing the information set 𝕽 from the received image, given as:

$$\begin{array}{c} R=\{(o_{i},h_{i})|i\in m\} \end{array}$$ (1)

where, *o*_*i*_ is the size of the *i* ^*th*^ object, and *h*_*i*_ is its respective height from the baseline of the image, received from the *i* ^*th*^ WMSN node camera. Only one object per image will be considered. As such, the object closest to the WMSN node camera will be preferred. This object will either have maximum size or minimum height. An example is illustrated in Fig 2, where an image received from node *i* contains two objects *o*_*i*_ and $o_{i}^{\prime }$. After prioritizing the objects based on their size and height, *o*_*i*_ is ultimately selected.

**Fig 3:**
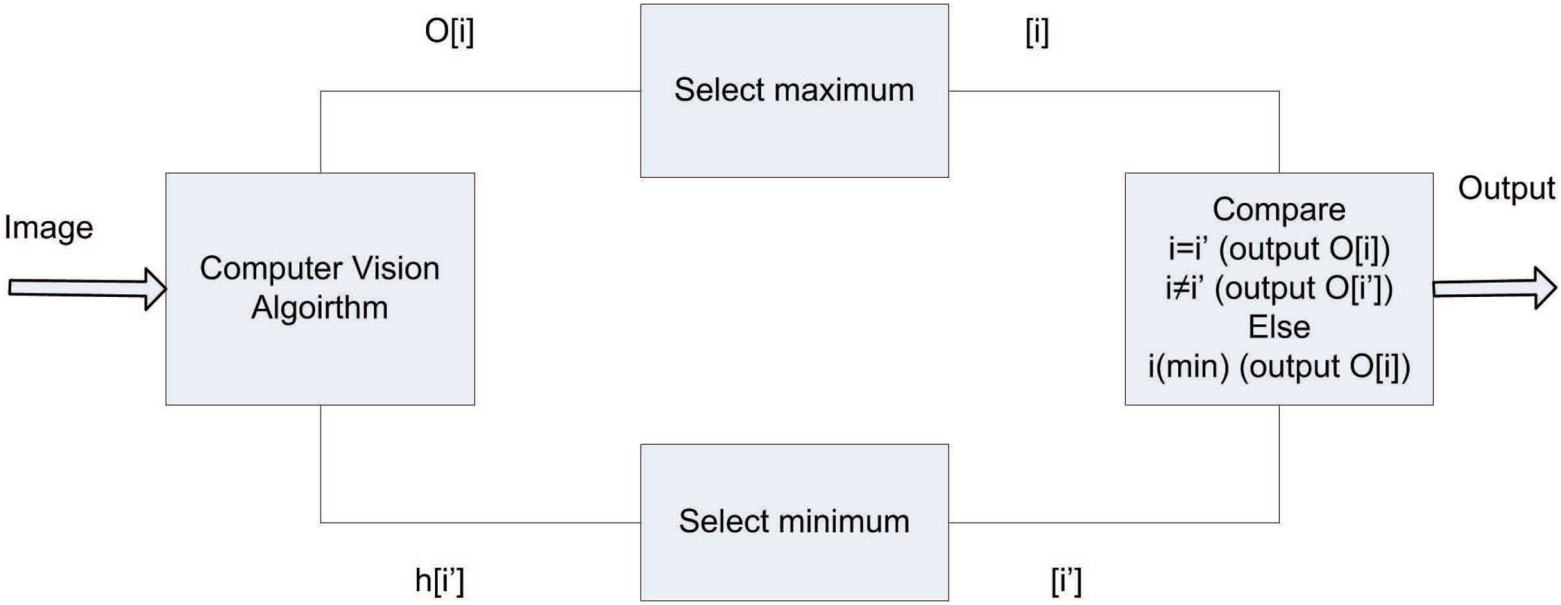
Selecting objects for initial distance estimation.

Before discussing various cases, it is important to describe the indexing process of the various parameters used for prioritizing the objects. The object sizes are arranged such that the object with smallest index *i* is the object having maximum area with respect to other objects in the same image. The object height index *i*′ are arranged such as the smallest index *i*′correspond to the minimum height of the object from the base line. In the present case the objects with minimum heights are given priority however, the addition of object size parameter reliably select the objects in case of multiple objects sharing same height and size parameters. The selection process is shown in Fig 3, where three possibilities can arise in the entire process.

1. If the index *i* of a maximum object size and *i*′ of minimum object height are the same, then the object corresponding to index *i* is selected.
2. If the index *i* of a maximum object size and *i*′ of minimum object height are not the same, then the object corresponding to index *i*′ is selected.
3. If there are multiple objects with same maximum object size, or with same minimum object height, then priority is assigned to the object having the least index *i* amongst the participating indices.

**Fig 4:**
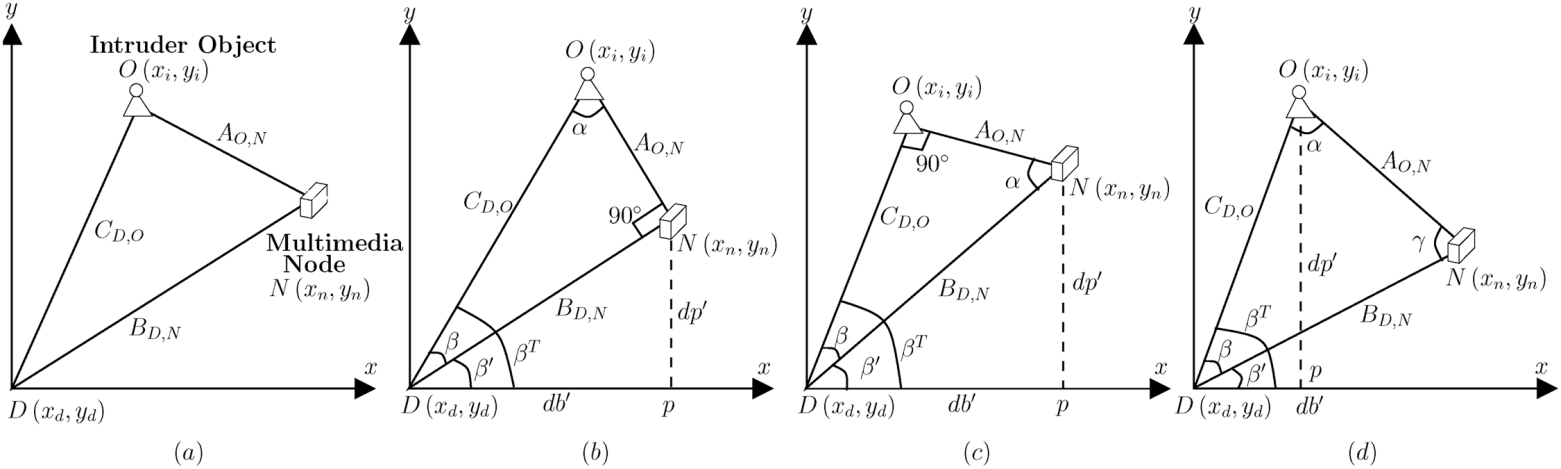
(a) Referential frame for estimating location of an object. (b) Case where object distance from the sink node is greater as compared to monitoring WMSN node *C*_*D*,*O*_ > *B*_*D*,*N*_. (c) Case where object distance from the sink node is smaller as compared to monitoring WMSN node *C*_*D*,*O*_ < *B*_*D*,*N*_. (d) Case where object distance from the sink node is equal to the monitoring WMSN node *C*_*D*,*O*_ = = *B*_*D*,*N*_.

Upon selection of an appropriate object from a received image, the distance between the object and the WMSN node is then estimated. A referential frame is designed for this purpose, as shown in Fig 4(a). The sink at location *D*(*x*_*d*_, *y*_*d*_) is treated as the origin point. The distance between the sink and the WMSN multimedia node would already have been estimated using the DPAI algorithm [21].

The distance *A*_*O*,*N*_ between the object *O*(*x*_*i*_, *y*_*i*_) and the WMSN multimedia node *N*(*x*_*n*_, *y*_*n*_) is estimated from the image received at the sink node using PCA. For this, a predefined matrix *M* of *n* objects bearing size variations obtained at various distances between 1 upto 10 feet is defined:

$$\begin{array}{c} M=[\begin{array}{ccc} S_{11} & \ldots & S_{1n}\\ \vdots & \ddots & \vdots \\ S_{n1} & \ldots & s_{nn} \end{array}], \end{array}$$ (2)

where, each element *S*_*i*,*j*_ represents the size of the *ith* object at a distance of *j* ft. The mean *μ*, variance *var*, and covariance *cov* of the vectorized form *S* = [*S*_11_, *S*_12_, …, *S*_*nn*_] of matrix *M* are given as:

$$\begin{array}{c} \mu =\frac{1}{n^{2}}\sum_{i}^{n}\sum_{j}^{n}S_{i,j} \end{array}$$ (3)

$$\begin{array}{c} var\left(S\right)=(S_{i}-\mu ) \end{array}$$ (4)

$$\begin{array}{c} cov\left(S\right)=\frac{1}{n}\sum_{i}^{n}(S_{i}-\mu )\cdot {\left(S_{i}-\mu \right)}^{T} \end{array}$$ (5)

Then, the eigen vectors for the covariance matrix are calculated and arranged in descending order as: *E* = [*E*_*g*1_, *E*_*g*2_, …, *E*_*gn*_]. Of these, the first twenty larges eigen vectors are selcted. A new feature set *F* is then obtained as:

$$\begin{array}{c} F=var{\left(S\right)}^{T}\cdot E_{1\ldots 20} \end{array}$$ (6)

For a given object size *o*_*i*_ the variance *P*, feature vector *U* and distance *d*_*i*_ can be given as:

$$\begin{array}{c} P=o_{i}-\mu \end{array}$$ (7)

$$\begin{array}{c} U=P^{T}\cdot E_{1\ldots 20} \end{array}$$ (8)

$$\begin{array}{c} d_{i}=\text{norm}F_{i}-U \end{array}$$ (9)

Finally, the minimum distance from the set *d*_*i*_ is computed as:

$$\begin{array}{c} d_{\min i}=\{d_{i}\}=A_{O,N} \end{array}$$ (10)

where, *d*_min*i*_ corresponds to the closest match for *U* in *F*_*i*_ in the given vectorized matrix *S*. Thus, *d*_min*i*_ = *A*_*O*,*N*_ is taken as the distance between source WMSN multimedia node and the object. After computing *A*_*O*,*N*_, the coordinates of object *O* can be obtained as:

$$\begin{array}{c} (x_{i},y_{i})=(x_{n},y_{n})+A_{O,N} \end{array}$$ (11)

Finally, the distance *C*_*D*,*O*_ between the sink node *D* and the object *O* can be computed using the distance formula:

$$\begin{array}{c} C_{D,O}=\sqrt{{\left(x_{d}-x_{i}\right)}^{2}+{\left(y_{d}-y_{i}\right)}^{2}} \end{array}$$ (12)

Once all the distances *B*_*D*,*N*_, *A*_*O*,*N*_ and *C*_*D*,*O*_ are computed, the object orientation *β* ^*T*^ is then calculated. For this purpose, the edges of triangle formed by the coordinates of WMSN multimedia node *N*, object *O*, and sink node *D* are analyzed. For the case where distance *C*_*D*,*O*_ is equal to *B*_*D*,*N*_, triangle Δ*DOP* (See Fig 4d) can be used to compute the orientation *β* ^*T*^ as:

$$\begin{array}{c} \beta^{T}=\{\begin{array}{cc} \begin{array}{c} \tan^{-1}\frac{dp^{\prime }}{db^{\prime }}\\ \beta +\beta^{\prime } \end{array} & \begin{array}{c} C_{D,O}==B_{D,N}\\ otherwise \end{array} \end{array} \end{array}$$ (13)

For cases where the two distances are un-equal, orientation *β* ^*T*^ is computed as a sum of angles *β* and *β*′ of two constituent triangles; Δ*DNO* and Δ*DPN* (See Fig 4b and 4c). These are given as:

$$\begin{array}{c} \beta =\{\begin{array}{cc} \begin{array}{c} \tan^{-1}\frac{A_{O,N}}{B_{D,N}}\\ \tan^{-1}\frac{A_{O,N}}{C_{D,O}} \end{array} & \begin{array}{c} C_{D,O}>B_{D,N}\\ C_{D,O}<B_{D,N} \end{array} \end{array} \end{array}$$ (14)

$$\begin{array}{c} \beta^{\prime }=\{\begin{array}{cc} \begin{array}{c} \tan^{-1}\frac{dp^{\prime }}{db^{\prime }}\\ \tan^{-1}\frac{db^{\prime }}{dp^{\prime }} \end{array} & \begin{array}{c} C_{D,O}>B_{D,N}\\ C_{D,O}<B_{D,N} \end{array} \end{array} \end{array}$$ (15)

## Simulation Results

**Fig 5:**
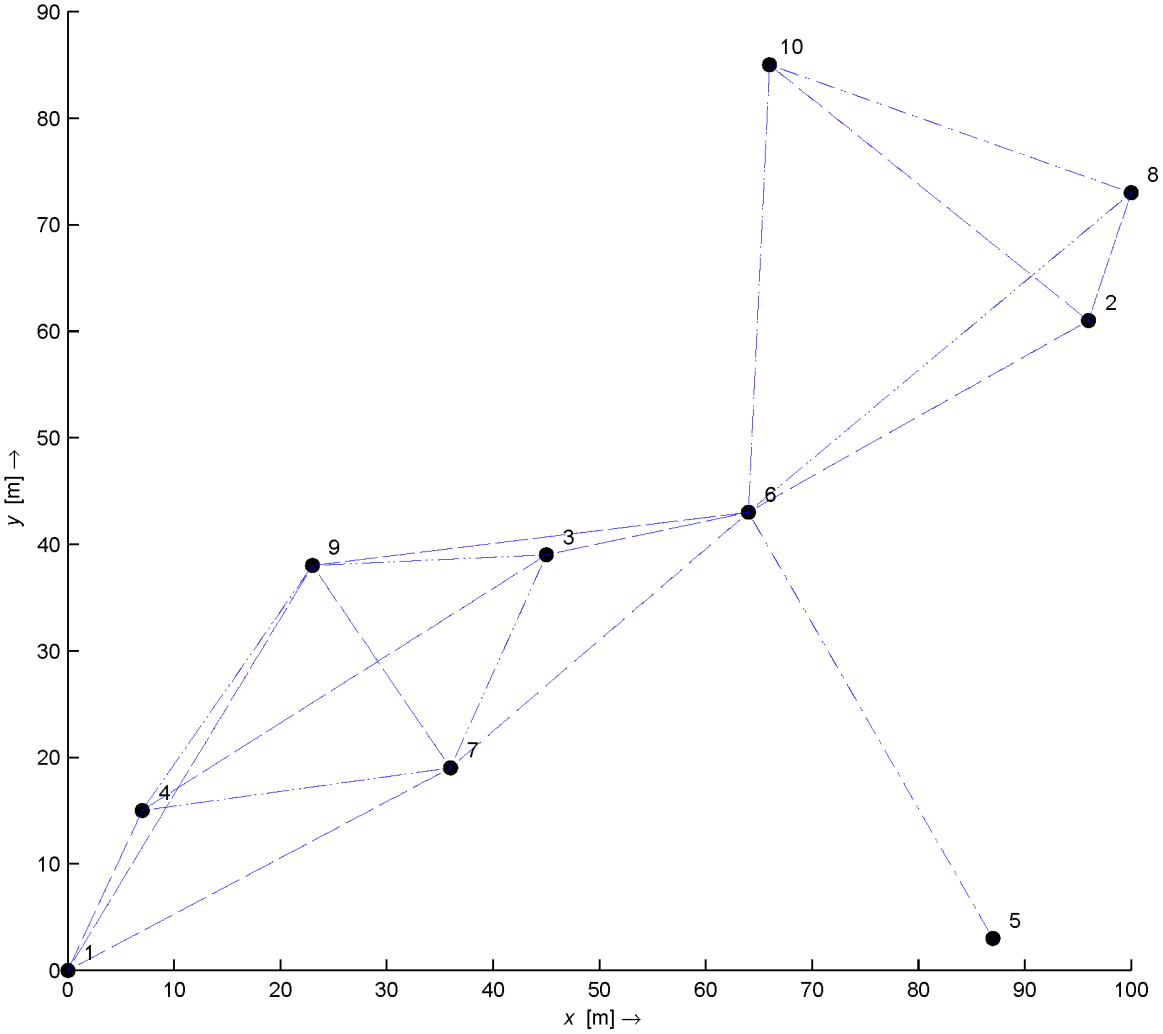
Network Map.

To analyze the performance of the proposed method with, we randomly deployed 10 WMSN sensor nodes, including the destination node in a 100 × 100 *meter* field as shown in Fig 5. The radio range is set to 25 meters to increase probability of maximum number of neighbors for each node. The nodes are first localized using the DPAI method [21]. After localization, every sensor node transmits an LL1 sub-band image (acquired after a 2D-DWT) of the monitored scene to the sink node. The sink node then performs a computer vision algorithm to find and extract any objects of interest. If found, these are recorded in set 𝕽 along with their size and height from image baseline. For multiple objects in the same image, a priority selection process is performed to select only one candidate object. This information is then fed to the PCA algorithm, which, based upon its predefined database, provides a distance estimate for the given size.

**Fig 6:**
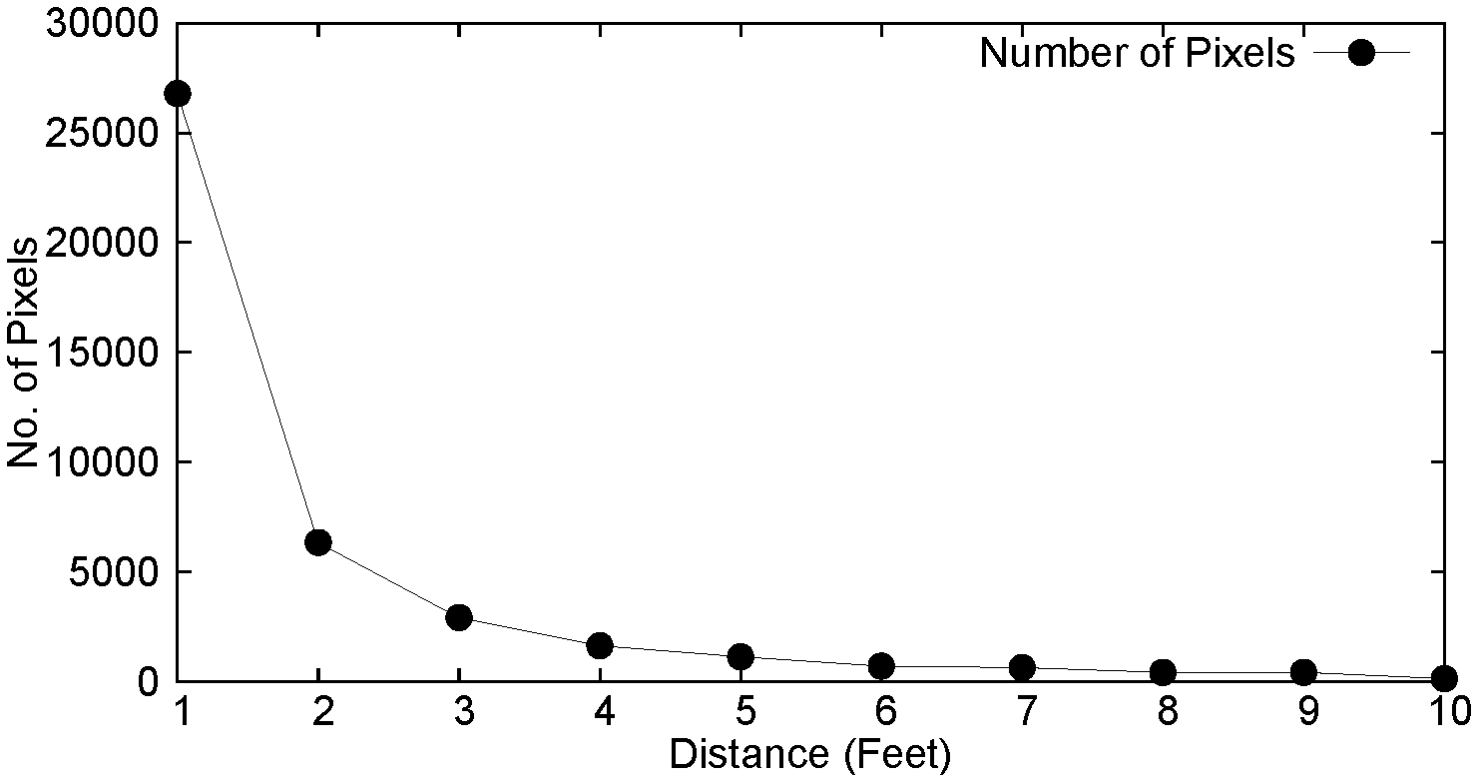
Variation of Object Size with Distance.

To further investigate the geometry of the object in the received frame, the variation in size of the isolated objects with respect to distance is studied. For a specific object, the object size varies exponentially with distance from the camera as shown in the Fig 6. For the farthest distances, the object size can be observed to be of almost 1 pixel.

To calculate the distance between the object and the sink node, the PCA algorithm is trained initially with 10 different objects. During the training phase, each object image is taken at a distance of 1 to 10 feet from the camera. From these images, the respective object size is extracted. A marix *M* of the objects is then constructed Eq (2). For *M*, a feature set *F* and distance *d*_*i*_ is then computed using Eqs (6)–(9). For an unknown object size, the minimum distance between the sink node and WMSN node is estimated using Eqs (11) and (12).

### Distance Estimation between WMSN node and Object

Tables 1 and 2 show the computed object distance and size using PCA. This is also compared to a regression based technique and Oztarak et al. [28]. For simplicity, variation of a single object’s size with respect to a distance from 1, upto 10 feet is taken. As seen in Table 1, the frequency of error in the PCA based localization is reported as 1/10, compared to other localization techniques. This error is attributed towards uncertainty in the object’s size at the farthest distance. *Therefore, PCA technique computes the same distance at 9 and 10 feet. Table 2 shows object size calculation in term of pixels of the object given that the distance of the object is in ft. In this case, the error in PCA based technique is exactly 0 as compared to other*.

**Table 1:** Distance estimation and associated error.

Distance (feet)	Object Size	PCA	Regression	Oztarak et. al	Error (PCA)	Error Regression	Error Oztarak et. al.
1	27328	1	1.06347	0.50065	0	0.06347	0.49935
2	6399	2	6.06899	0.97627	0	4.06899	1.02373
3	3114	3	6.85465	1.35593	0	3.85465	1.64407
4	1647	4	7.20551	1.87744	0	3.20551	2.12256
5	1189	5	7.31505	2.2704	0	2.31505	2.7296
6	746	6	7.421	2.87139	0	1.421	3.12861
7	574	7	7.46214	3.14926	0	0.46214	3.85074
8	465	8	7.48821	3.61582	0	0.51179	4.38418
9	364	9	7.51236	4.0678	0	1.48764	4.9322
10	288	10	7.53054	4.64891	1	2.46946	5.35109

**Table 2:** Estimation of object size and its associated error.

Distance (feet)	Object Size	PCA	Regression	Oztarak et. al	Error (PCA)	Error Regression	Error Oztarak et. al.
1	27328	27328	83678	32235	0	56349.92238	4907.80618
2	6399	6399	27671	7999	0	21271.86142	1600.37498
3	3114	3114	28336	4017	0	31450.19953	903.83225
4	1647	1647	84343	2110	0	85990.26049	463.32888
5	1189	1189	140350	1482	0	141539.32145	293.72081
6	746	746	196357	928	0	197103.3824	182.64202
7	574	574	252364	742	0	252938.44336	168.70721
8	465	465	308372	582	0	308836.50431	117.22418
9	364	364	364379	457	0	364742.56527	68.38182
10	288	288	420386	356	0	420673.62622	68.38182

**Fig 7:**
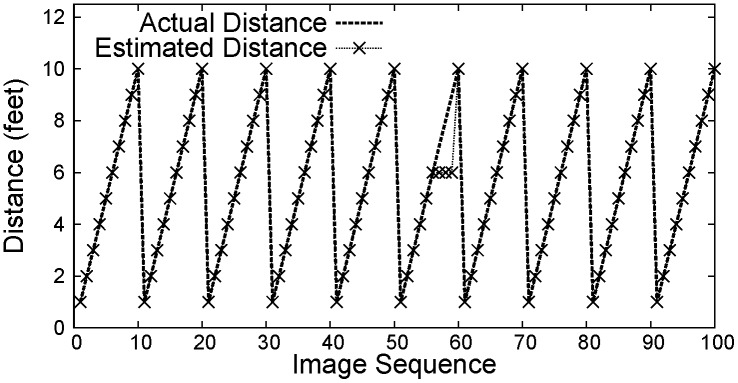
PCA based localization.

**Fig 8:**
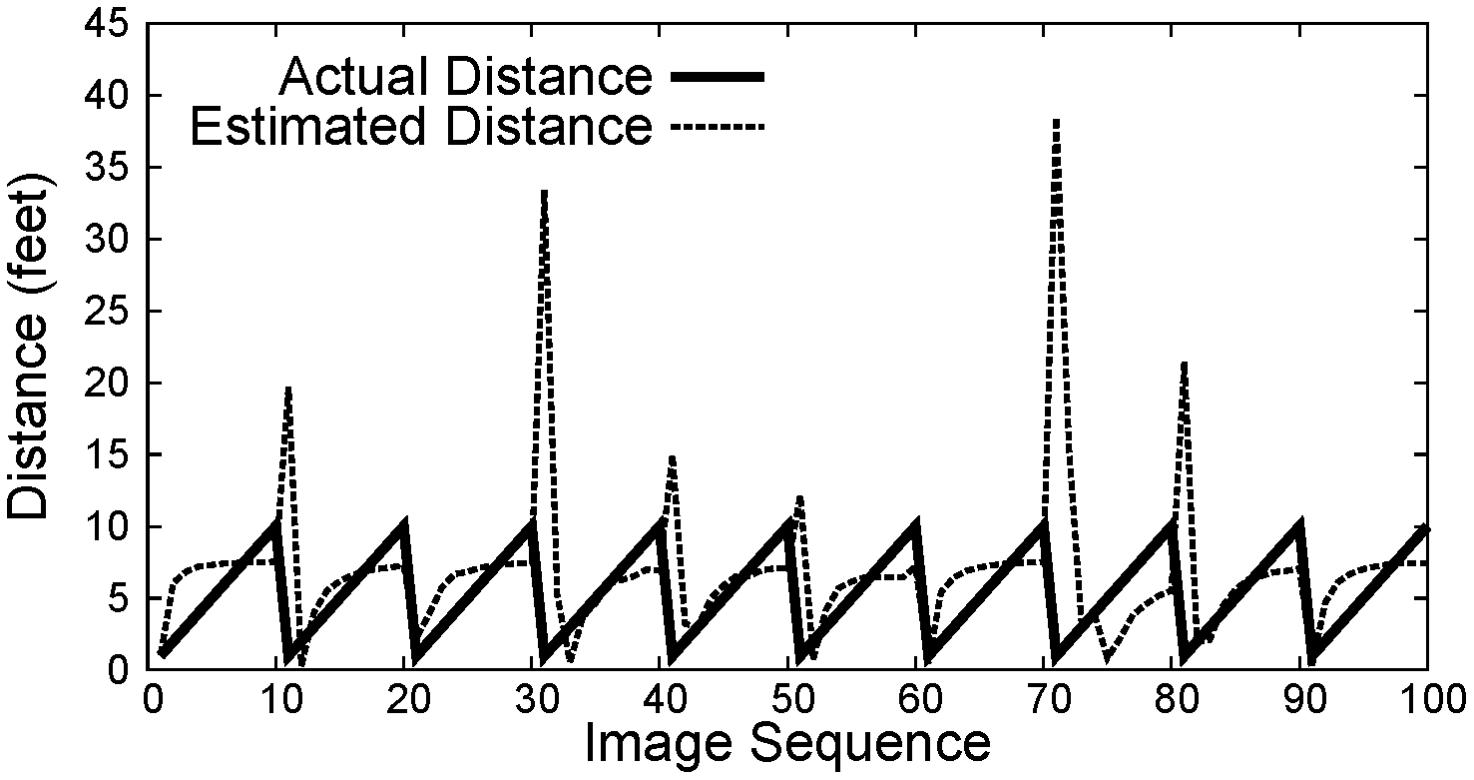
Regression based localization.

**Fig 9:**
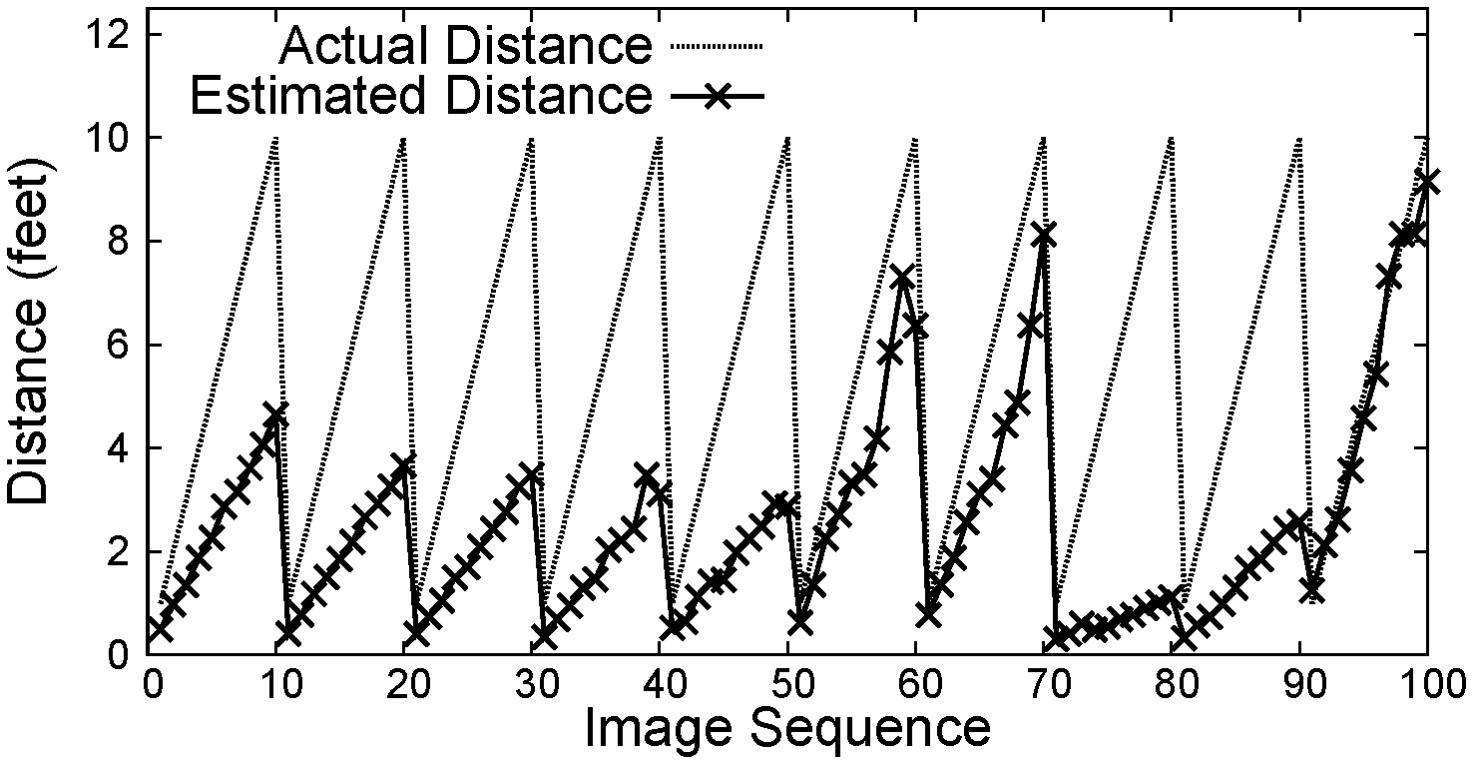
Localization results by Oztarak. et. al. [28].

**Fig 10:**
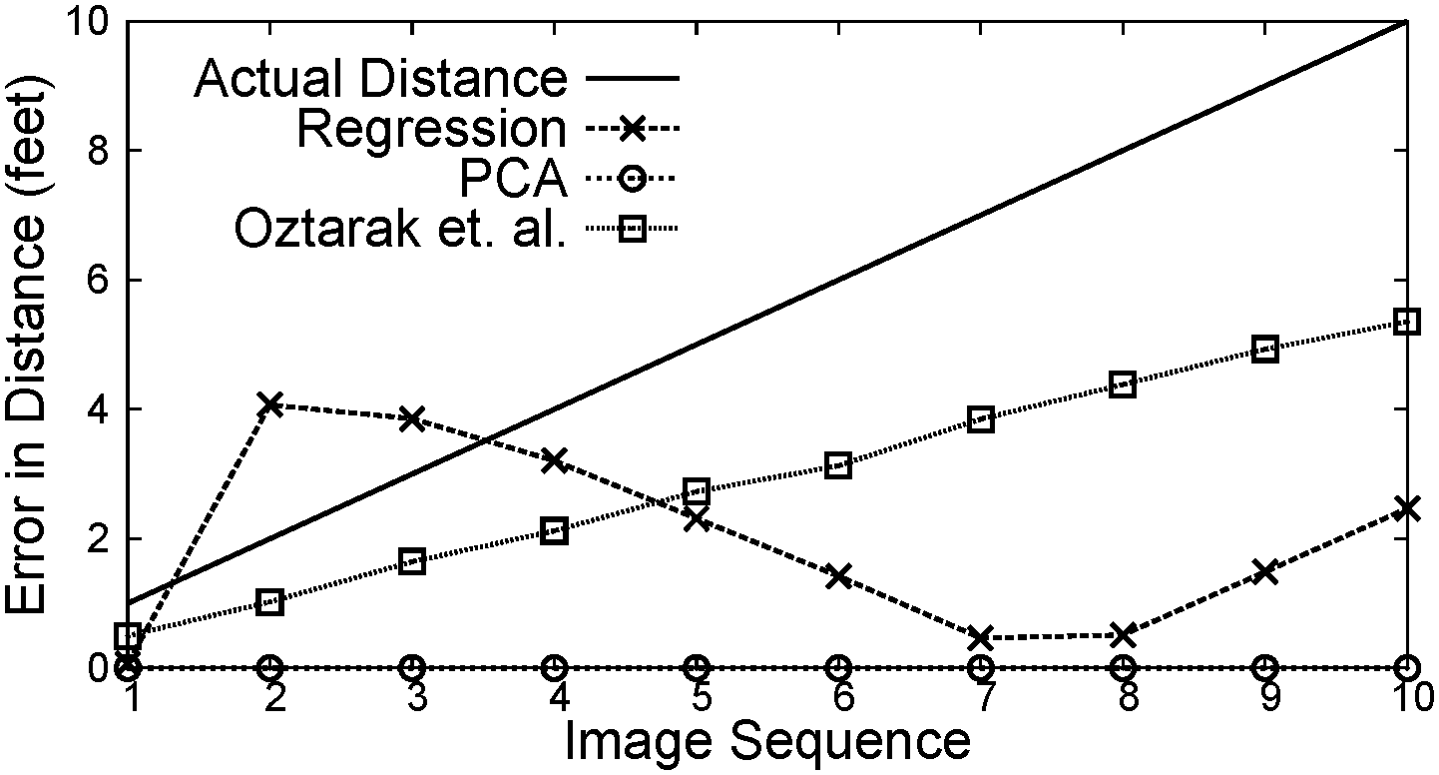
Error in distance estimation between regression, PCA, and Oztarak. et. al. based localization methods.

Figs 7–9 show the distance between the WMSN node and the object using PCA, Regression and Oztarak et al. [28] based localization techniques. In Fig 7, the error between the actual distance and the distance estimated by PCA technique is almost zero. Fig 10 show the error in distance estimation between Regression, PCA and Oztarak et. al. [28] based localization technique. Again, it can be observed that the error in PCA based technique is almost zero.

**Fig 11:**
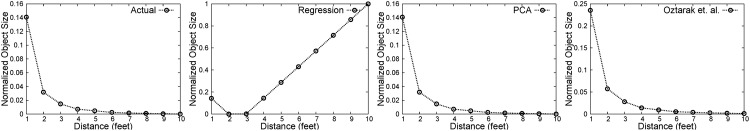
Normalized object sizes against distance (in feet).

The efficiency *η* of the proposed method is calculated as:

$$\begin{array}{c} \eta =\frac{T-T_{noError}}{T}\times 100, \end{array}$$ (16)

where *T* represents the total observations, and *T*_*noError*_ represents the number observations where an error was reported. With this, it can be observed that the percentage efficiency of calculating distance between the object and the source WMSN node is 40% using the method by Oztarak et al. [28], 59% using regression, and 99% using our PCA based approach. Fig 11 shows the complete result of the normalized object size variation with distance.

**Fig 12:**
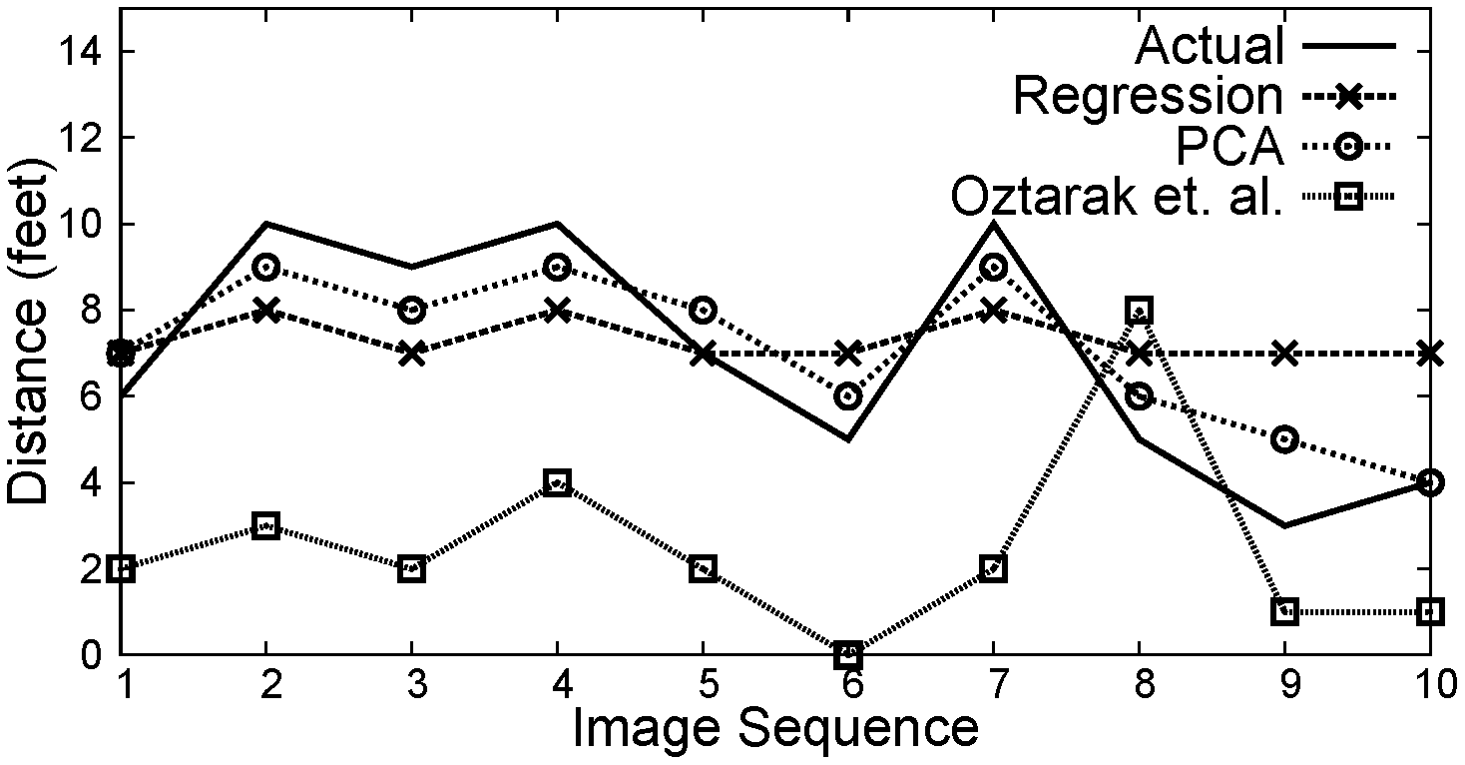
Distance estimation under received noisy measurement.

**Fig 13:**
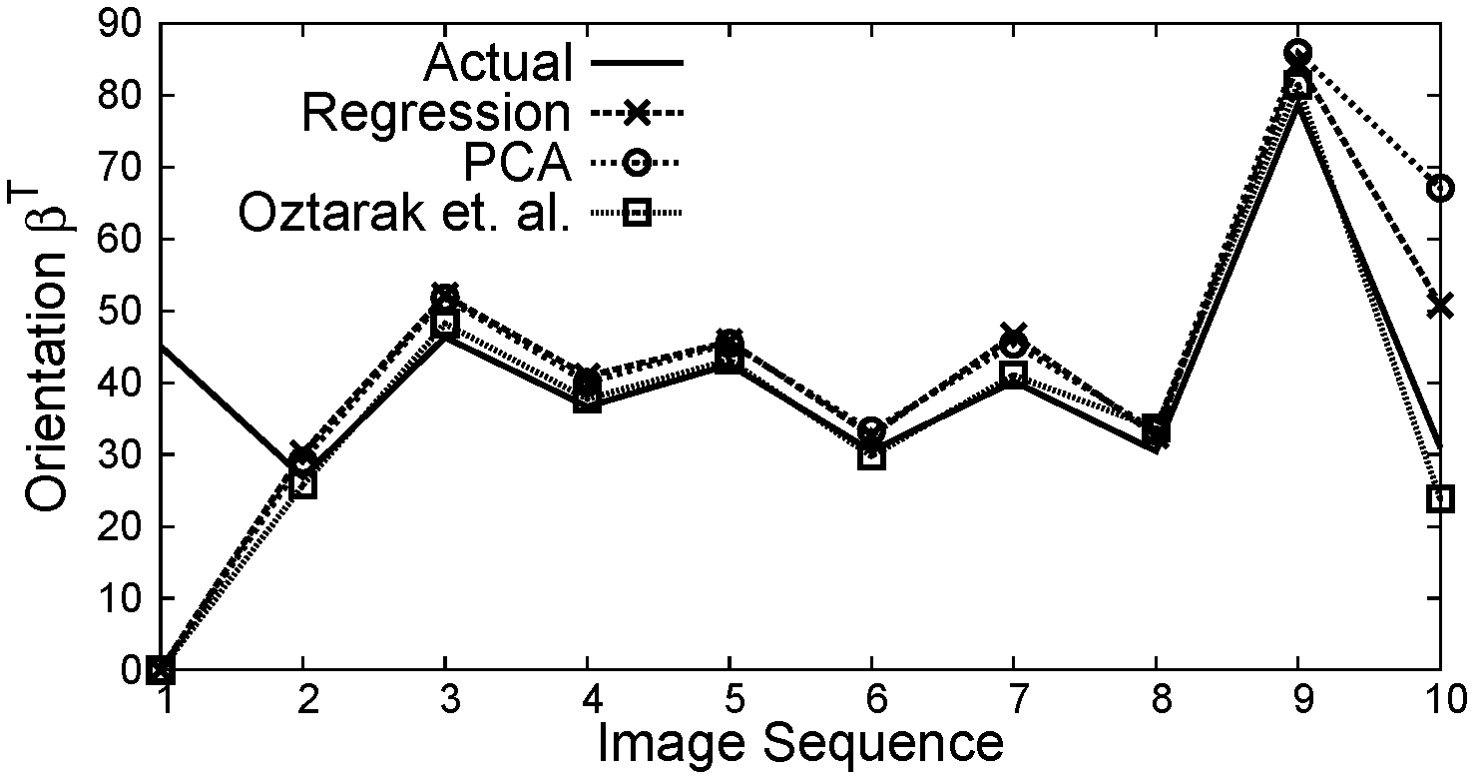
Object orientation *β* ^*T*^ obtained at sink node from various image sources *i*.

Fig 12 shows the distance estimation under noisy measurements, where it can be observed that the distance estimated using PCA based technique provides a statisfactory result as compared to Regression and Oztarak et. al. [28] based techniques. Fig 13 shows the result of object orientation with respect to sink node. Here, it can be observed that the orientation using all three localization methods is close to the actual orientation.

Table 3 reports the comparison of PCA, Regression and Oztarak et. al. [28] techniques in terms of various parameters such as size and camera orientation. To calculate the efficiency of these techniques, a tolerance of 2 feet for distance estimation and 200 pixels for size estimation has been considered. The ratio *ρ* is given as:

$$\begin{array}{c} \rho =\frac{T_{error}}{T_{noError}} \end{array}$$ (17)

**Table 3:** Comparison of various techniques.

Technique	Object Actual Size	Camera Parameters	*μ* in calculating distance	Distance Error Ratio *ρ*	*μ* in calculating size	Size Error Ratio *ρ*
PCA	No	No	99%	0.01	100%	0
Regression	No	No	59%	0.69	2%	49
Oztarak et. al.	Yes	Yes	40%	1.5	61%	0.64

**Fig 14:**
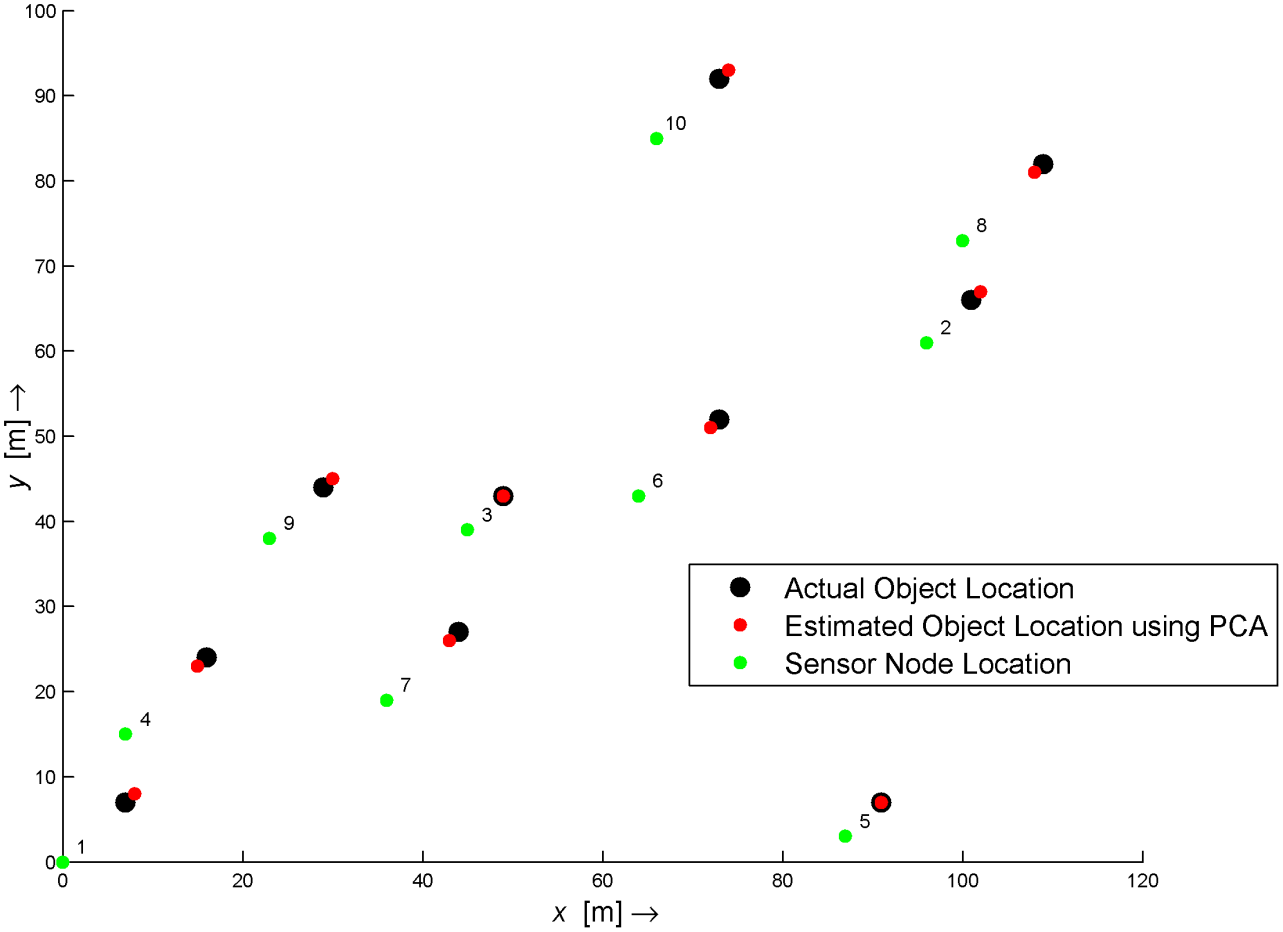
Final Result.

Table 4 shows the estimated object location by using PCA, Regression and Oztark et. al. [28] based technique. As can be depicted in Table 4 the error is reduced to 1 feet by using PCA based technique.Fig 14 shows the final result in the Network Map.

**Table 4:** Localization comparison of different schemes.

	Actual		PCA		Regression		Oztarak et. al	
Sensor #	X	Y	X	Y	X	Y	X	Y
1	6	6	7	7	7	7	1	1
2	81	41	80	40	79	39	74	32
3	65	68	64	67	63	66	58	60
4	86	64	85	63	84	62	79	56
5	107	98	108	99	107	98	102	92
6	102	60	103	61	104	62	97	55
7	64	54	63	53	62	52	56	45
8	102	60	103	61	104	62	104	58
9	15	75	17	77	19	79	13	72
10	10	6	10	6	13	9	7	2

## Conclusion

This manuscript presents a method for object localization in wireless multimedia sensor networks that uses range free localization, machine learning, and computer vision based techniques. Object localization is an important component of many popular applications of WMSN. As such the first step is to localize the WMSN nodes. The image acquisition process then begins where all images are processed before being delivered to the sink node. The main objective of this processing is to reduce network bandwidth, and is performed by application of a 2D Discrete Wavelet Transform 2D Discrete Wavelet Transform composes the image into various sub-bands of varying sizes and quality. The sink employs a PCA based technique to localize the object. Node orientation and object geometry are taken into account in the entire process. Simulation results report 99% efficiency and an error ratio of 0.01 (around 1 ft) when compared to other popular techniques.

## Supporting Information

S1 Dataset 10 Images of a Rechargable battery taken at distance of 1 up to 10 feet’s. (RAR)

S2 Dataset 10 images of a 12 × 8 Inches box taken at a distance of 1 upto 10 feet’s. (RAR)

S3 Dataset 10 images of a 4 × 8 Inches box taken at a distance of 1 upto 10 feet’s. (RAR)

S4 Dataset 10 images of a brown color bag taken at a distance of 1 upto 10 feet’s. (RAR)

S5 Dataset 10 images of a black color bag taken at a distance of 1 upto 10 feet’s. (RAR)

S6 Dataset 10 images of an umbrella taken at a distance of 1 upto 10 feet’s. (RAR)

S7 Dataset 10 images of a Paint Can taken at a distance of 1 upto 10 feet’s. (RAR)

S8 Dataset 10 images of a Cloth bin taken at a distance of 1 upto 10 feet’s. (RAR)

S9 Dataset 10 images of a shopping bag taken at a distance of 1 upto 10 feet’s. (RAR)

S10 Dataset 10 images of a Bunch of Electrical wires taken at a distance of 1 upto 10 feet’s. (RAR)

S1 Documentation Documentation about the dataset. (DOCX)
