# Supplementary material for: Improved Object Localization Using Accurate Distance Estimation in Wireless Multimedia Sensor Networks
Source: PLoS One. 2015 Nov 3;10(11):e0141558. doi: 10.1371/journal.pone.0141558 (PMC4631602; doi:10.1371/journal.pone.0141558)
Supplement: S1 Documentation — (DOCX) [file pone.0141558.s011.docx]

This data contains 10 images of the following objects taken from 1 up to 10 feet’s.

1. A 6 Inches Rechargeable battery
2. A 12 x 8 Inches box
3. 4 x 8 Inches box
4. A bag (Brown color)
5. A bag (Black Color)
6. An umbrella
7. A paint Can
8. A cloths bin
9. A shopping bag
10. Bunch of Electrical Wires

This data set can be used to estimate the location of an unknown object at a distance of 1 up to 10 feet from a source node. The objects can be extracted from these images by using any computer vision algorithm. The extracted objects size and its position in the image can be used as a reference for the estimation of objects with unknown distance from the source node.
